# Heterologous Immunization With Defined RNA and Subunit Vaccines Enhances T Cell Responses That Protect Against *Leishmania donovani*

**DOI:** 10.3389/fimmu.2018.02420

**Published:** 2018-10-17

**Authors:** Malcolm S. Duthie, Neal Van Hoeven, Zachary MacMillen, Alessandro Picone, Raodoh Mohamath, Jesse Erasmus, Fan-Chi Hsu, Dan T. Stinchcomb, Steven G. Reed

**Affiliations:** Infectious Disease Research Institute, Seattle, WA, United States

**Keywords:** leishmania, protein, vaccine, RNA, adjuvant

## Abstract

The rapid generation of strong T cell responses is highly desirable and viral vectors can have potent CD8^+^ T cell-inducing activity. Immunity to leishmaniasis requires selective T cell responses, with immunization schemes that raise either CD4 or CD8 T cell responses being protective in small animal models. We have defined the leishmaniasis vaccine candidate recombinant fusion antigens, LEISH-F2 and LEISH-F3+, that when formulated in a stable emulsion with a Toll-like receptor (TLR) 4 agonist, induce protective CD4^+^ T cell responses in animal models as well as providing therapeutic efficacy in canine leishmaniasis and in clinical trials in leishmaniasis patients. We used the genetic sequences of these validated vaccine antigens to design RNA vaccine constructs. Immunization of mice with the RNA replicons induced potent, local innate responses that were surprisingly independent of TLR7 and activated antigen-presenting cells (APC) to prime for extremely potent antigen-specific T helper 1 type responses upon heterologous boosting with either of the subunit vaccines (recombinant antigen with second generation glucopyranosyl lipid A in stable oil-in-water emulsion; SLA-SE). Inclusion of RNA in the immunization schedule also generated MHCI-restricted T cell responses. Immunization with LEISH-F2-expressing RNA vaccine followed later by subunit vaccine afforded protection against challenge with *Leishmania donovani*. Together, these data indicate the utility of heterologous prime-boost immunization schemes for the induction of potent antigen-specific CD4 and CD8 T cell responses for protection against intracellular pathogens.

## Introduction

It is critical that we develop effective interventions to counteract infectious agents that continue to emerge (1). RNA vaccine technology has demonstrated its ability to induce antibody responses and, thus, the potential to generate rapid responses to several emerging pathogens (2–5). Relative ease in the design and manufacture of nucleic acid-based vaccines also suggests the potential for inexpensive and somewhat generic production, and robust manufacturing processes have been developed for some targets (4). Among the strategies employed to enhance the immunogenic nature of nucleic-acid vaccines has been adaptation of the nucleic acid vector to permit targeting of the innate immune system to enhance subsequent adaptive responses, and manipulation of the delivery systems to allow efficient transfection of host cells *in vivo*. Although viral vectors such as alpha- and lentivirus-based constructs also potently activate T cells, less is known with regard to how RNA vaccine technology can be used for the induction and characterization of T cell responses against intracellular pathogens.

*Leishmania donovani* is an important human pathogen that can manifest visceral leishmaniasis (VL). It is estimated that up to 90% of humans infected with *L. donovani* remain in an asymptomatic state, with an effective antigen-specific T cell response critical for containing the infection and preventing advancement to VL (6). Experimental *Leishmania* infection of mice has broadened our understanding of helper T cells during an infection and has allowed dissection of the Th1/Th2 paradigm (7–9). Additionally, because protection is conferred by Th1 cells in resistant strains and susceptibility is induced by Th2 cells in susceptible strains, these models have revealed genetic mechanisms involved in disease development (7, 10–12). These experimental findings are highly pertinent because CD4 T cells of asymptomatic, infected individuals produce IFNγ in a *L. donovani* antigen-specific manner (13–20). There is also evidence that interplay with CD8 T cells can also participate in protection in both experimental and physiological situations (17, 21, 22). Given that they are well described, *Leishmania* infection models provide controlled experimental systems with which to evaluate T cell-inducing vaccines (23). To date, we have produced several defined subunit vaccines consisting of recombinant fusion proteins appropriately formulated with adjuvant that elicit protective Th1 responses (24–26).

Considering the importance of antigen-specific T cells in the control of several emerging infectious diseases, we decided to use the LEISH-F2 and LEISH-F3+ fusion proteins as known antigenic targets to determine if we could rapidly produce vaccine candidates capable of raising protective T cell responses. We developed an alphavirus-based RNA replicon expressing the F2 and F3+ genes (F2-RNA and F3+ RNA, respectively) then assessed their ability to complement the activity of a defined subunit (recombinant protein with second generation glucopyranosyl lipid A in stable oil-in-water emulsion; SLA-SE) vaccines for the rapid and potent generation of antigen-specific T cell responses.

## Materials and methods

### RNA replicon

A plasmid encoding the 5′ and 3′ untranslated regions and nonstructural genes of Venezuelan equine encephalitis virus (VEEV) strain TC-83 was utilized as the replicon backbone, as previously described (27). Gene sequences for LEISH-F2 and LEISH-F3+ constructs were codon optimized, synthesized, and cloned into pUC57 vector at NotI and SphI sites by Genscript (Piscataway, NJ). Lyophilized DNA was reconstituted in distilled water and digested with NotI-SphI then cloned into Tc83 replicon. Replicon DNA was linearized by enzymatic digestion firstly with NotI then proteinase K, followed by phenol chloroform treatment and ethanol precipitation. Plasmid was transcribed using MEGAscript® T7 Transcription Kit (Invitrogen, Carlsbad, CA) followed by capping with NEB Vaccinia Capping System. Expression of the insert was confirmed by Western blot. Briefly, 2 ul of cell lysate was loaded and run on a tris-glycine gel and then transferred to nitrocellulose paper. The transferred nitrocellulose paper was then incubated with anti-F2 rabbit serum in 5% dry milk phosphate buffered saline-Tween (PBST) followed by detection using goat anti-rabbit IgG (Southern Biotech, Birmingham, AL 4050-05).

### Cell stimulations

HEK293-Blue-TLR2, -TLR3, -TLR4, -TLR7, or null cells (all from InvivoGen, San Diego, CA) were maintained in DMEM+GlutaMax cell culture medium containing 10% FCS, 1% penicillin and streptomycin, 50 ug/ml neomycin, and selective antibiotics (HEK-Blue™ Selection, Blasticidin or Zeocin; InvivoGen). Cells were untreated or treated with the mixture of Lipofectamine™ 3,000 (Invitrogen) and F2 or control RNA constructs. For stimulation, cells were seeded into 96-well plates at a concentration of 2.5–5 × 10^5^ cells per ml in the HEK-Blue™ detection medium (InvivoGen) then incubated with corresponding TLR agonists. Activation induced expression of SEAP (secreted embryonic alkaline phosphatase) and were measured spectrophotomerically at OD650 nm.

### Mice

Female C57BL/6 mice (purchased from Charles River Laboratories, Wilmington, MA) and TLR7–/– (purchased from Jackson Laboratories, Bar Harbor, ME) were maintained in specific pathogen-free conditions and in accordance with animal procedures approved by the IDRI institutional animal care and use committee. Mice entered experiments at 6–8 weeks of age.

### Draining lymph node assay

Mice were injected with 20 μl vaccine into calf muscle. The RNA vaccine was prepared to provide a total of 10 μg dose. SLA-SE was injected at 5 μg per injection to serve as a positive control for immune activation. At various times after injection, the draining lymph node was excised and single cell suspensions prepared. Mononuclear cells were enumerated using a ViaCount assay with a PCA system (Guava Technologies, Hayward, CA).

### Immunizations

Mice were immunized by intramuscular injection of vaccines into the thigh.

Recombinant fusion proteins LEISH-F2 and LEISH-F3+ were constructed by aligning the individual gene sequences as a single product that was then cloned and expressed in *E. coli* as previously described (28, 29). Affinity-purified protein fractions were analyzed by sodium dodecyl sulfate-polyacrylamide gel electrophoresis (SDS-PAGE) and quantified using the BCA protein assay (Pierce, Rockford, IL). Endotoxin levels were measured by Limulus Amebocyte Lysate QCL-1000 assay (Lonza Inc., Basel, Switzerland) and each was < 100 EU/mg protein. The recombinant antigen-containing vaccine (F2 or F3+ with SLA-SE) were prepared to provide a total of 5 μg protein and 5 μg SLA per injection. The putative compact binding of SLA to TLR4 appears to result in a cytokine profile more reminiscent of TRIF-dependent signaling compared with the alternative TLR4L glucopyranosyl lipid adjuvant (GLA) (30). RNA constructs were prepared to provide a total of 10 μg dose. Each vaccine was delivered in a total volume of 100 μl, split to a volume of 50 μl in each thigh. Homologous and heterologous prime-boost regimens with these two vaccines involved a priming event followed 3 weeks later with a booster immunization.

### Antigen-specific antibody responses

Blood was collected from the retro-ortital sinus and serum prepared. Antigen-specific IgG responses were detected by enzyme linked immunosorbent assay (ELISA). Briefly, ELISA plates (Nunc, Rochester, NY) were coated with 1 μg/ml antigen in 0.1 M bicarbonate buffer and blocked with 0.1% BSA-PBS. Then, in consecutive order and following washes in PBS/Tween, serially diluted serum samples, anti-mouse IgG-HRP (Southern Biotech, Birmingham, AL) and ABTS-H_2_O_2_ (Kirkegaard and Perry Laboratories, Gaithersburg, MD) were added to the plates. Plates were analyzed at 405 nm (EL_X_808, Bio-Tek Instruments Inc, Winooski, VT). Endpoint titer was determined as the last optical density (OD) value greater than a threshold determined by sera from saline-immunized control mice.

### Antigen-specific cytokine secretion

Spleens were removed 4 weeks after the final immunization and single cell suspensions prepared. Cells were cultured at 2 × 10^5^ cells per well in duplicate in a 96-well plate (Corning Incorporated, Corning, NY) in RPMI-1640 supplemented with 5% heat-inactivated FCS and 50,000 Units penicillin/streptomycin (Invitrogen). The antigen-specific recall response was determined by incubating the cells with 10 μg/ml recombinant protein or MHCI-restricted peptides for 3 days before collection of the culture supernatant. Cytokine (IL-5 and IFNγ) content in the supernatant was measured by ELISA according to the manufacturers' instructions (eBioscience, Inc., San Diego, CA).

### Flow cytometry

To determine the immune cell composition of draining lymph nodes (DLN), single cell suspensions were prepared and incubated with Fc receptor block (clone2.4G2) before incubation at room temperature for 30 min with the following antibodies: anti-CD4 (clone RM4-5), anti-CD8a-FITC (clone 53-6.7), anti-CD11b (clone M1/70), anti-CD11c (clone N418), anti-CD19 (clone 1D3), anti-F4/80 (clone BM8), and anti-Ly6G (clone 1A8). Expression of selected surface molecules was determined by incubation with anti-CD69 (clone H1.2F3) and anti-CD80 (clone 16-10A1). Antibodies were purchased from BD Biosciences, eBioscience, BioLegend or Tonbo Biosciences.

Antigen-specific T cell memory responses generated by vaccination were determined following incubation of spleen cells with 10 μg/ml F2 recombinant protein. Cells were cultured at 1 × 10^6^ cells per well in duplicate in a 96-well plate (Corning Incorporated, Corning, NY) in RPMI-1,640 supplemented with 5% heat-inactivated FCS and 50,000 Units penicillin/streptomycin (Invitrogen) for 18–20 h in the presence of GolgiStop (BD Bioscience). Each sample was incubated with Fc receptor block (clone2.4G2) before incubation at room temperature for 30 min with the following antibodies: anti-CD4 (clone RM4-5), anti-CD8a-FITC (clone 53-6.7), and anti-CD44 (clone IM7). Expression of selected cytokines was determined by incubation with anti-IFNγ (clone XMG1.2), anti-IL-2 (clone JES6-5H4), anti-CD154 (clone MRI), anti-TNFα (clone MP6-XT22), anti-IL-5 (clone TRFK5), and anti-Granzyme B (NGZB). Antibodies were purchased from BD Biosciences, eBioscience, BioLegend or Tonbo Biosciences. Data were acquired using Fortessa (BD Biosciences) and analyzed with FlowJo software (FlowJo, LLC).

### *Leishmania* challenge of mice

Mice were infected by injection of 1 × 10^6^ *L. donovani* (MHOM/SD/00/1S-2D) into the retro-orbital sinus. Four weeks later livers were harvested and homogenized. DNA was extracted from homogenate using QIAmp DNA mini kits (Qiagen) and quantified using Nanodrop UV-Vis spectrophotometer (ND-1000). *L. donovani* DNA was then detected by real-time PCR using primers for L42486 (forward, 5′- GCGACGTCCGTGGAAAGAA-3′; and reverse, 5′- GGCGGGTACACATTAGCAGAA-3′) with FAM reporter sequence (5′- CAACGCGTATTCCC-3′) that detects a 203-bp genomic repeat region specific to *Leishmania* species (NCBI Blastn). Mouse Gapdh FAM (Life Technologies) was used as an internal reference control. The number of parasite per μl of DNA was determined by extrapolating the crossing points (Cps) of each sample against a standard curve generated with known quantities of parasites, then burdens expressed as parasites per organ.

### Statistical analyses

Statistical analyses were conducted using one-way analysis of variance and Dunnett's or Tukey's multiple comparison test used to compare two groups. Statistical significance was considered when the *p*-values were < 0.05.

## Results

### F2 RNA induces NF-κB translocation via TLR7 engagement

**Figure 1:**
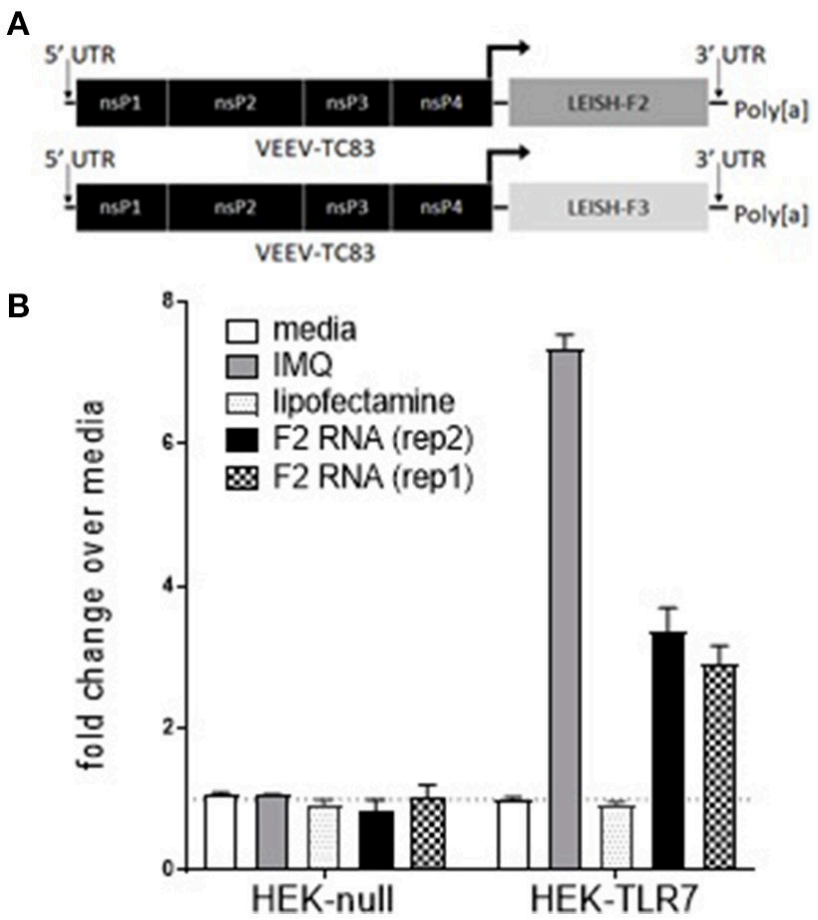
F2-RNA replicons engage intracellular TLR7. In **(A)**, schematic depictions of the F2-RNA and F3+-RNA replicons are shown. In **(B)**, HEK293-Blue cells and HEK293-Blue expressing human TLR7 were incubated with imquimod (IMQ), lipofectamine, or lipofectamine and two different production runs of F2 RNA vaccine construct (rep1 and rep2). Activation induced expression of SEAP was measured at OD650 nm. Data were generated for each condition in triplicate and are shown as mean and SE fold-change over media alone wells.

To determine if F2-RNA could promote activity through known innate immune receptors, HEK293-Blue cells expressing various human Toll-like receptor (TLR) were incubated with the RNA vaccine construct. The only tested condition that generated the induction of SEAP under the IFN-β promoter fused with NF-κB and AP-1 binding sites was when F2-RNA was incubated with HEK293-TLR7 cells that had been treated with Lipofectamine™ (Figure 1 and data not shown). These data indicate that F2 RNA can engage TLR7 within the cell to initiate the transcription of NF-κB-regulated genes.

### Early immune activation following inoculation of F2-RNA

**Figure 2:**
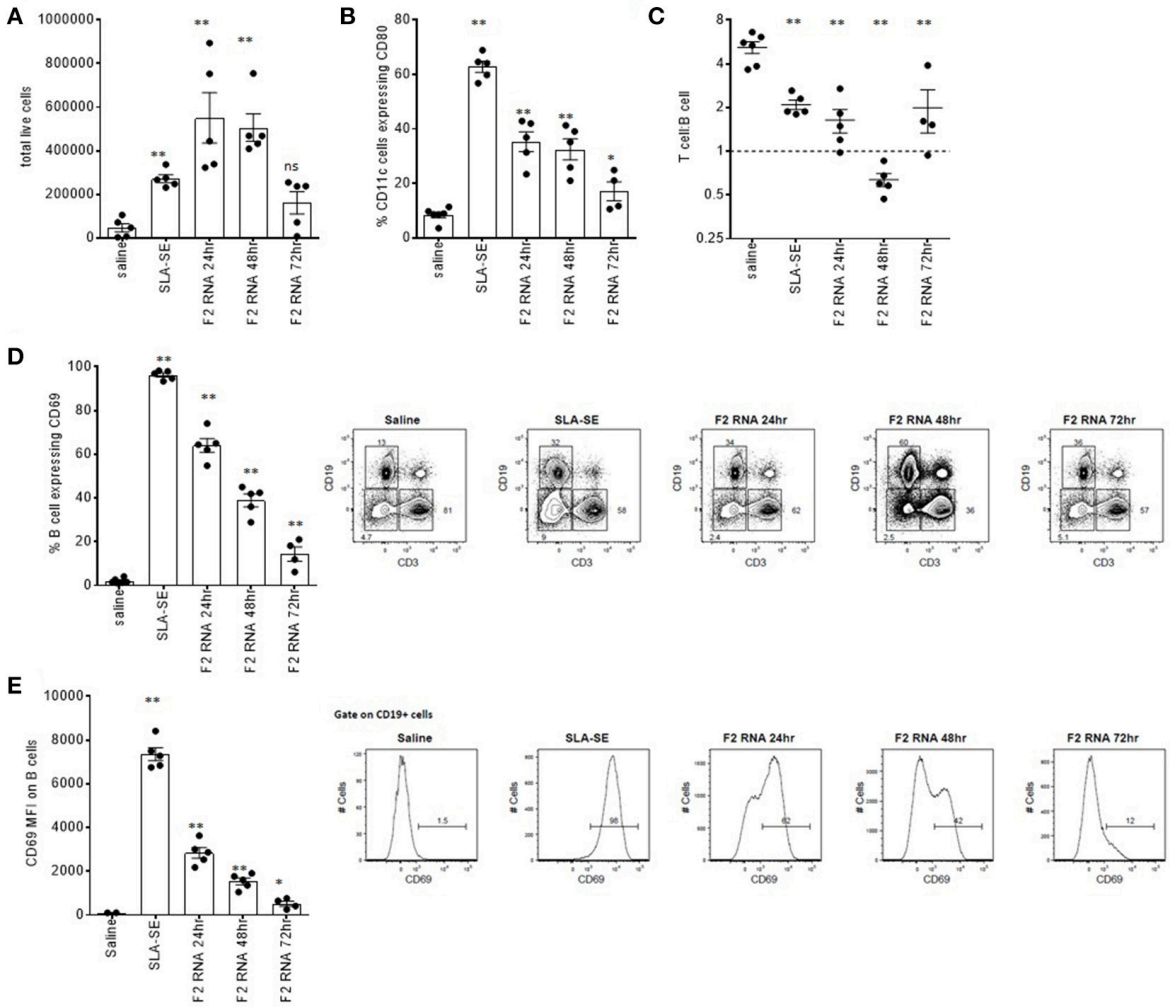
Rapid local immune activation following inoculation of RNA-F2. C57BL/6 mice were injected with 20 μl vaccine into calf muscle. The RNA vaccine was prepared to provide a total of 10 μg dose. SLA-SE was injected at 5 μg per injection to serve as a positive control for immune activation. At various times after injection, the draining lymph node was excised and single cell suspensions prepared and **(A)** enumerated. Cells were incubated with fluorescently labeled antibodies then subjected to flow cytometry to determine **(B)** activation state (CD80 expression) on CD11c-expressing cells; **(C)** T cell (CD3) to B cell (CD19) ratio; **(D,E)** activation state (CD69 expression) on CD19-expressing B cells. Each point depicts the data from a single sample (*n* = 5 per group), while the horizontal and vertical bars show the mean and SEM, respectively. Data are representative of results obtained in 2–3 independent experiments. ns = not significant, and * and ** = *p*-value < 0.05 and < 0.01, respectively, when comparing the indicated group against the saline injected control group.

We previously demonstrated that mice receiving TLR4 agonist in stable emulsion (SE) had measurable alterations in their draining lymph nodes, including upregulation of early activation markers (31). Injection of F2-RNA into the calf muscle similarly caused a dose-dependent enlargement of the draining lymph node and a measurable increase in DLN cell numbers 24 and 48 h following injection (Figure 2A and data not shown). Flow cytometry revealed the upregulation of the costimulatory CD80 molecule on CD11c^+^ DC, as well as an inversion of the ratio of T cells to B cells within the node 48 h after injection (Figures 2B,C). A large proportion of the B cells within the DLN of F2-RNA immunized mice expressed CD69, albeit at lower levels than mice treated with SLA-SE (Figure 2D). Both the proportion of B cells expressing CD69 and the level of CD69 were highest 24 h after injection and waned over time (Figures 2D,E). Thus, F2-RNA is capable of activating innate immune responses *in vivo*, generating an environment capable of inducing antigen-specific T cell responses.

**Figure 3:**
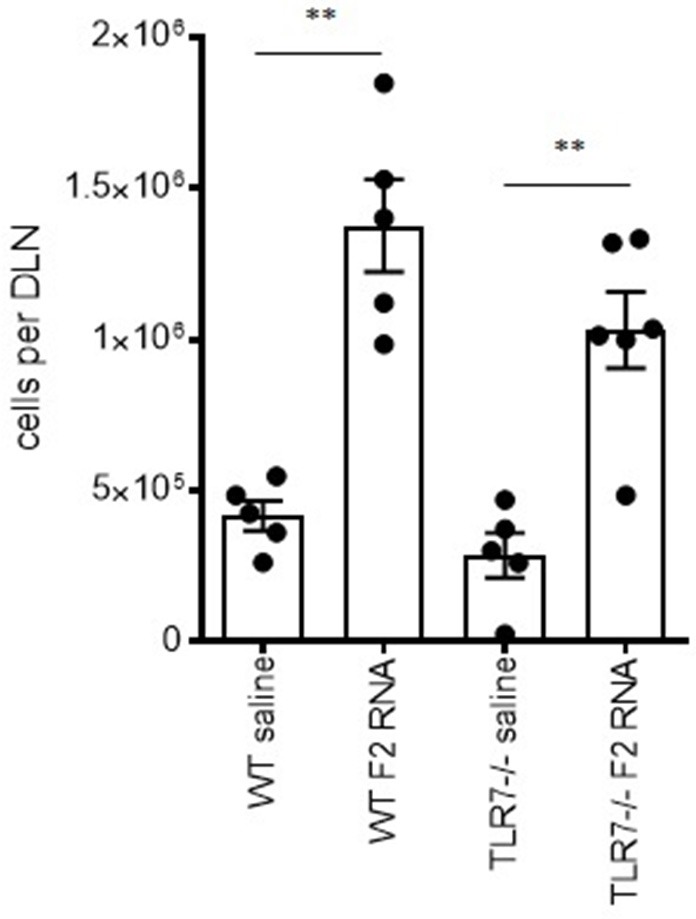
Local immune activation following inoculation of RNA-F2 is not critically dependent upon TLR7. Wild type and TLR7–/– C57BL/6 mice were injected with 20 μl vaccine into calf muscle. The RNA vaccine was prepared to provide a total of 10 μg dose. Twenty-four hours after injection, the draining lymph node was excised and single cell suspensions prepared and enumerated. Each point depicts the data from a single animal, while the horizontal and vertical bars show the mean and SEM, respectively. Data are representative of results obtained in 2-3 independent experiments. ns, not significant and **p-value < 0.01, respectively, when comparing the indicated group against the pertinent saline injected control group.

To assess the importance of the *in vitro* finding indicating TLR7 engagement, we injected either saline or F2-RNA into wild type and TLR7–/– mice then compared the subsequent DLN cell numbers. Immunization caused a significant increase in cell numbers within the draining lymph nodes from both mouse genotypes (Figure 3). When considered alongside the *in vitro* data generated with knock-in cells, these *in vivo* data indicate that, while involved in the response, TLR7 is not critical for the early immune activation by the F2-RNA vaccine.

### Heterologous prime-boost immunization schemes generate potent antigen-specific IFNγ secretion

**Figure 4:**
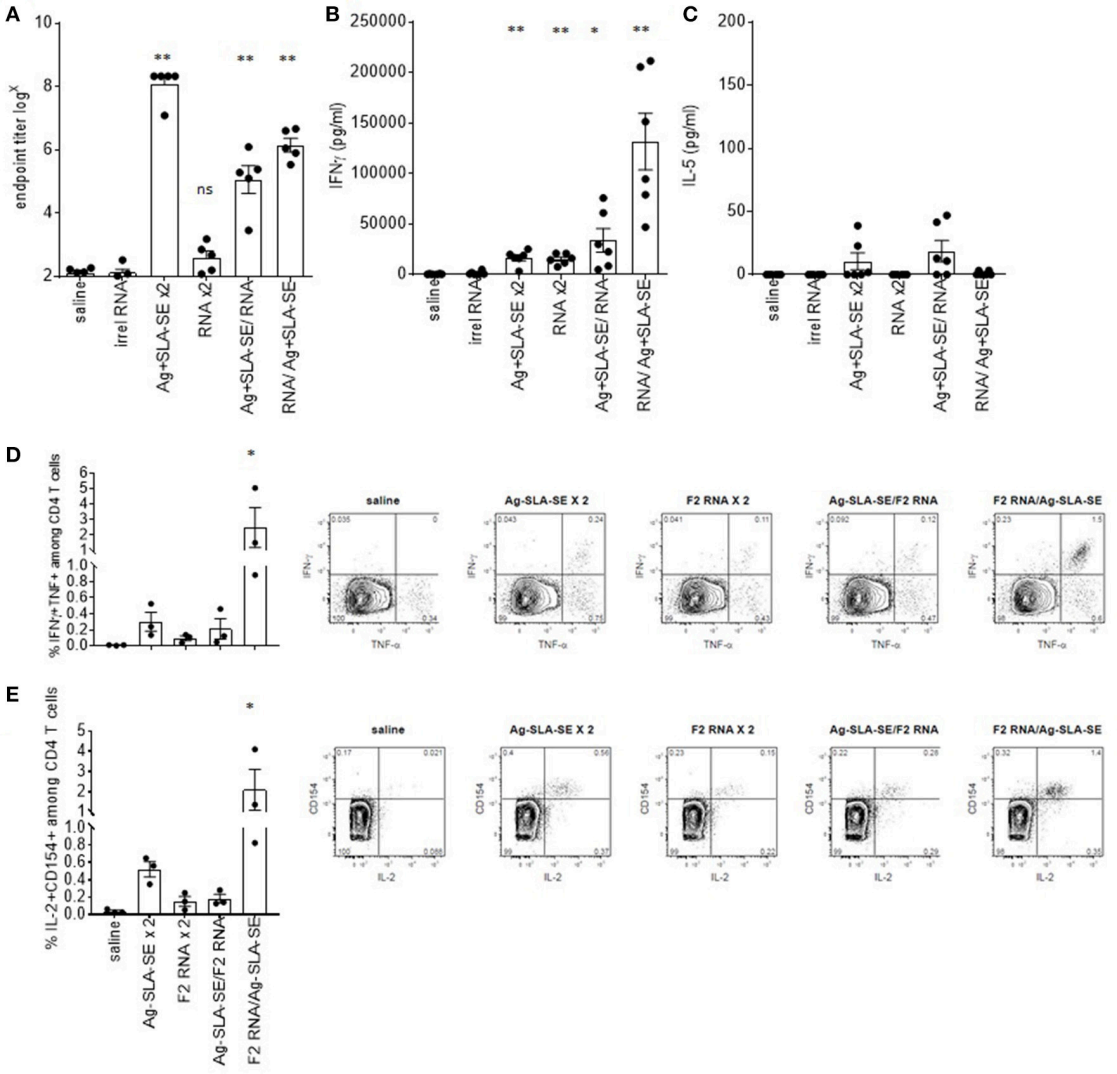
Dichotomous generation of humoral and cellular immunity by subunit and RNA vaccines. C57BL/6 mice were immunized with the indicated homologous or heterologous regimen of vaccines. Four weeks after the boost, blood was collected and serum prepared, and spleens were removed and single cell suspensions prepared. In **(A)**, the magnitude of the antigen-specific F2 IgG response was determined by dilution of each serum sample in ELISA until negative results were observed. Each point depicts the data from a single sample (*n* = 5 per group), while the horizontal and vertical bars show the mean and SEM, respectively. Data are representative of results obtained in 3 independent experiments. Spleen cells were incubated with the F2 protein, then **(B)** IFNγ or **(C)** IL-5 concentrations in the culture supernatants was determined by ELISA. Cells were also subjected to flow cytometry to determine the relative proportion of CD4 T cells simultaneously producing **(D)** IFNγ and TNF or **(E)** IL-2 an CD154. Data are shown as mean and SEM, *n* = 3–5 per group, and are representative of results obtained in 3 independent experiments. ns = not significant, and * and ** = *p*-value < 0.05 and < 0.01, respectively, when comparing the indicated group against the saline injected control group.

To compare the ability of F2-RNA and subunit vaccines to induce immune responses, both antigen-specific antibody and cytokine production were evaluated. Homologous prime-boost with F2+SLA-SE generated an extremely high antigen-specific IgG response (Figure 4A). This contrasted with the antibody response in the sera of mice immunized with F2-RNA that was detectable at only very low titers (Figure 4A). When mice were administered with both vaccines in heterologous prime/boost schemes, intermediate levels of antigen-specific antibodies at levels that could essentially be attributed to the single inoculation of F2+SLA-SE were detected (Figure 4A and data not shown). Similarly, a single immunization with a mixture of F2-RNA/F2+SLA-SE raised responses equivalent to the F2+SLA-SE immunization alone.

While a large quantity of IFNγ was secreted from cells from mice that received homologous immunizations with F2+SLA-SE, this was still lower than the extremely high amounts of IFNγ secreted from cells from mice primed with F2-RNA then later boosted with F2+SLA-SE (Figure 4B). Measurement of cytokines secreted fromcells incubated with F2 protein generally indicated the preferential secretion of IFNγ over IL-5 (Figures 4B,C). Taken together, these data indicate that although the regimen of F2-RNA priming, F2+SLA-SE boosting did not modify antibody responses, it did propagate potent antigen-specific CD4 T cells.

### Antigen-specific IFNγ secretion is from both CD4 and CD8 T cells

To directly evaluate antigen-specific T cell responses, memory CD44^hi^ CD4 T cells were identified among spleen cells following incubation with the F2 antigen. Activated antigen-specific cells, as indicated by CD154 (CD40L) expression and production of IL-2, were induced by all of the immunization regimens (Figure 4E; all *p*-value < 0.05 vs. saline group). Homologous prime-boost with F2+SLA-SE generated a more robust CD4 T cell memory response than homologous prime-boost with F2-RNA as revealed by a greater proportion of cells or producing IFNγ and TNF or producing IL-2 and expressing CD154 (Figures 4D,E). The heterologous prime-boost strategy involving priming with F2-RNA followed by a boost with protein and SLA-SE generated a substantial population of F2-specific CD4 T cells that was much larger than even that induced by the homologous prime-boost with F2+SLA-SE. These data demonstrate the even though the F2-RNA on its own did not raise a very large CD4 T cell response, it strongly primed cells for the promotion of a potent CD4 T cell response when further stimulated with F2+SLA-SE.

**Figure 5:**
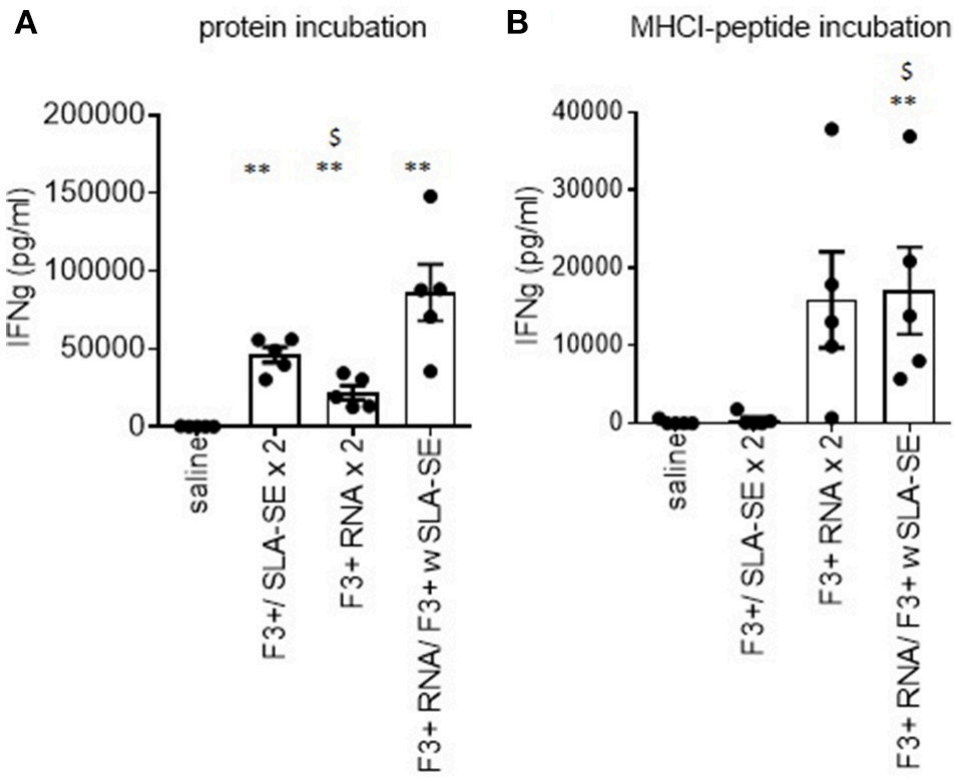
Differential generation of CD4 and CD8 T cell responses by subunit and RNA vaccines. C57BL/6 mice were immunized with the indicated homologous or heterologous regimen of vaccines incorporating the LEISH-F3+ antigen or sequence. Four weeks after the boost, spleens were removed and single cell suspensions prepared. Spleen cells were incubated with **(A)** F3+ protein or **(B)** a pool of MHCI-restricted peptides derived from F3+. IFNγ concentration in the culture supernatants was determined by ELISA. Data are shown as mean and SEM, *n* = 5 per group, and are representative of results obtained in 3 independent experiments. ns = not significant, and ** = *p*-value < 0.01, respectively, when comparing the indicated group against the saline injected control group. $ = *p*-value < 0.05, when comparing the indicated group against the F3+ with SLA-SE immunized group.

To extend the observations made with F2 vaccines, we immunized mice with vaccines containing the F3+ antigen (25). Incubation of spleen cells from mice that were immunized by homologous prime-boost with F3+ with SLA-SE or a heterologous RNA prime, protein/adjuvant boost produced cells that secreted IFNγ upon incubation with protein (Figure 5A). Given that we have defined MHCI-restricted epitopes within LEISH-F3+, these vaccines also allowed us to evaluate if antigen-specific CD8 T cells could be generated. Cells from mice provided the RNA-based vaccines (either in a homologous or heterologous manner) produced IFNγ in response to the peptides representing F3+ MHCI-restricted epitopes (Figure 5B). This contrasted with cells from mice immunized with F3+ with SLA-SE construct that had not demonstrable response (Figure 5B). Thus, unlike homologous schemes, heterologous prime with F3+ RNA construct followed by a boost with F3+ protein with SLA-SE generated both a robust CD4 T cell memory response and a CD8 T cell response.

### Heterologous prime-boost immunization schemes generate protection against experimental *L. donovani* infection

**Figure 6:**
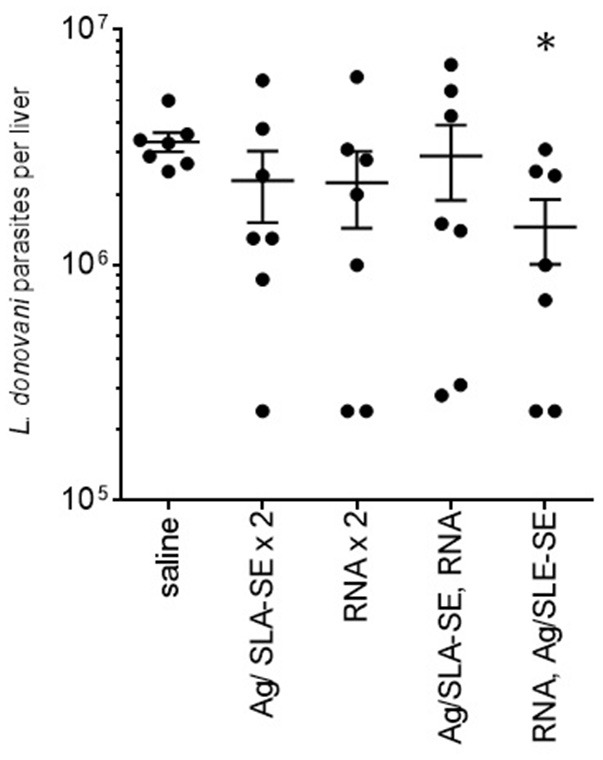
Protection against *L. donovani* infection by a heterologous prime-boost immunization regimen. C57BL/6 mice were immunized with the indicated homologous or heterologous regimen of F2-containing vaccines. Mice were infected by intravenous inoculation of *L. donovani* parasites 4 weeks after their final, or only, immunization. Four weeks after infection, livers were removed and parasite burden determined for each animal by PCR. Data are shown as mean and SEM, *n* = 7 per group. * = *p*-value < 0.05 when comparing the indicated group against the saline injected control group.

Having demonstrated that heterologous strategies using F2-RNA and F2+SLA-SE raised a mix of antigen-specific CD4 and CD8 T cell responses, we assessed if these responses could protect against *L. donovani* infection. Homologous (for a total of two) immunizations with either F2+SLA-SE or F2-RNA did not significantly reduced liver parasite burdens in *L. donovani*-infected mice (Figure 6). Parasite numbers were, however, significantly reduced in mice that were immunized with the heterologous F2-RNA prime/ F2+SLA-SE boost regimen (Figure 6, *p*-value = 0.015). Thus, shortened immunization schemes that included the RNA vaccine induced responses that were potent enough to protect against infection with an intracellular parasite.

## Discussion

In the face of emerging infectious diseases and drug-resistant pathogens, vaccines that can quickly induce protective responses are typically preferred. It is critical that rapid and scaled production procedures of such vaccines are developed. As a proof-of-concept for the rapid generation of a vaccine that mediates protection through T cells, we created F2 RNA with a goal of protecting against infection with *L. donovani*. Insertion of the F2 gene into an alphavirus replicon created F2 RNA that induced innate responses via engagement of intracellular TLR7 and promoted local immune responses with activation of APC. While immunization with F2 RNA alone elicited only a small population of antigen-specific Th1 cells, a heterologous immunization scheme involving priming with F2 RNA followed by boosting with a LEISH-F2+SLA-SE vaccine (recombinant antigen/ synthetic adjuvant) resulted in extremely potent Th1 responses and IFNγ secretion.

From their initial conception, by mimicking immunization with a live vaccine, nucleic acid vaccines, delivered virally, such as with viral replicon particles or similar systems have held promise as an effective way to induce T cell immunity. In particular, viral delivery of replicon RNA derived from the alphavirus genus has demonstrated potent CD8 T cell responses (32). However, dependence on viral proteins to mediate delivery of nucleic acids induces anti-vector immunity, precluding repeated use of the platform for other indications (33). To circumvent this concern, we focused on immunization with a naked RNA replicon derived from a vaccine strain of the alphavirus Venezuelan equine encephalitis virus (VEEV), TC-83, which has a long history in pre-clinical and, more recently, clinical development (32, 34, 35).

Clinical trials have demonstrated the safety and tolerability of nucleic acid vaccines (36, 37). The relative ease in the design and manufacture of nucleic acid-based vaccines suggests that they can be produced inexpensively and in a somewhat generic manner. Although we did not develop an RNA vaccine against a *de novo* target, by building upon a subunit vaccine with an already defined target we were able to use a RNA vaccine producing the same target to enhance immune responses and reduce the need for more than two immunizations. Coupling of the RNA and subunit vaccines in a heterologous prime boost scheme generated extremely potent CD4 T cell responses that removed the need for multiple immunizations with the subunit vaccine. Our data therefore imply that under conditions where the available quantities of a defined vaccine may be limited (i.e., pandemic outbreaks), the rapid generation of an RNA vaccine encoding the same target antigen could be rapidly generated as a means of filling the supply gap.

Interestingly, our data indicate that immunization with F2 RNA alone generated immune responses that were qualitatively different from the LEISH F2 subunit vaccine. Immunization with the subunit vaccine induced slightly larger Th1 responses and very much larger antibody responses than immunization with the F2 RNA alone, which generated low Th1 responses and only very weak antibody responses. These data indicate the important impact of the antigen production/ presentation platform has on immunity, and suggest that naked RNA immunization can be used for the preferential induction of T cell responses. Indeed, experiments with F3+ RNA vaccine allowed us to distinguish the preferential induction of antigen-specific CD8 T cell responses. Long term immunization experiments are required to determine how long these CD4 and CD8 T cell responses persist. Minimizing antibody generation may be beneficial in clinical or therapeutic situations where immune complexes can cause complications.

Our data demonstrate that although both RNA and subunit vaccines induced early changes in the cellular composition and activation state of the draining lymph node there were some distinguishing features. While F2+SLA-SE induced the majority of CD11c^+^ cells to upregulate CD80 and CD19^+^ cells to express high levels of CD69, these alterations were induced at lower levels consequent to F2 RNA injection. In contrast, F2 RNA caused a more pronounced adjustment of the T cell: B cell ratio within the draining lymph node than F2+SLA-SE. At present we can only speculate that these early differences contribute to dichotomous antigen-specific T cell responses elicited by each vaccine. Further evaluation of these early events and the cytokine profiles within the draining lymph node appear merited. Similarly, the relative contribution of either CD4 or CD8 T cells to protection against *Leishmania* infection could be delineated by further immune comparisons or use of gene-deficient mice.

A restrictive feature of naked RNA vaccines to date has been their disappointing potency, indicating that efforts to enhance immunogenicity are needed. Similar to other reports examining mRNA, our data indicate that the F2 RNA replicon engages TLR7 to activate the innate immune responses (38, 39). This activation leads to alterations in the cellular composition within the local lymph node. It is well documented that TLR7 signaling is compatible with a wide variety of innate receptors to enhance responses, suggesting the possibility of incorporating further agonists to provide an additive or synergistic impact of T cell responses (40). This would act in a manner similar to the enhancement of mRNA vaccine potency by administration with Flt3 ligand or RNA encoding GM-CSF (41, 42). It should also be noted that our study used naked RNA replicon (i.e., formulated only in a saline diluent) and a great deal of research is currently being conducted to formulate RNA in order to protect it from degradative enzymes as well as to enable its delivery across the cell membrane. Work has focused on encapsulation in liposomes and complexing with cationic polymers to both protect them from degradation and enhance cellular uptake (43, 44).

Together, our results demonstrate the rapid production of an RNA vaccine and further indicate the utility of RNA vaccines for the promotion of CD4 T cell responses. This development process has great appeal for the rapid provision of vaccines that can protect against newly emerging pathogens that lack clear treatment or control strategies.

## Author contributions

MD, NH, JE, DS, and SR designed experiments and contributed to manuscript writing. ZM, RM, JE constructed RNA replicons. ZM, AP, MD, and F-CH performed immunization and immunoassay experiments. MD, NH, JE, and F-CH performed data analyses.

### Conflict of interest statement

Steven Reed and Malcolm Duthie are co-inventors on a patent for leishmaniasis vaccine development. All remaining authors declare no competing interests.

## Abbreviations

APC: antigen presenting cell
ELISA: enzyme linked immunosorbent assay
GLA: glucopyranosyl lipid adjuvant
IFN: interferon
IL: interleukinp
RNA: ribonucleic acid
SE: stable emulsion; SLA
TLR: Toll-like receptor
TNF: tumor necrosis factor
VEEV: Venezuelan equine encephalitis virus
VL: visceral leishmaniasis.
